# Acteoside and isoacteoside alleviate renal dysfunction and inflammation in lipopolysaccharide-induced acute kidney injuries through inhibition of NF-κB signaling pathway

**DOI:** 10.1371/journal.pone.0303740

**Published:** 2024-05-15

**Authors:** Jing Lian, Yisheng Xu, Ji Shi, Pengpeng Liu, Yue Hua, Chao Zhang, Tianhang Ren, Guoming Su, Shizan Cheng, Zixuan Nie, Tianzhu Jia

**Affiliations:** 1 School of Pharmacy, Liaoning University of Traditional Chinese Medicine, Dalian, China; 2 Waters Technology (Beijing) Co., Ltd., Beijing, China; National Institutes of Health, UNITED STATES

## Abstract

Acute kidney injury (AKI) is a sudden loss of renal function with a high mortality rate and inflammation is thought to be the underlying cause. The phenylpropanoid components acteoside (ACT) and isoacteoside (ISO), which were isolated from *Cistanche deserticola* Y.C.Ma, have been reported to have preventive effects against kidney disorders. This study aimed to investigate the anti-inflammatory properties and protective mechanisms of ACT and ISO. In this investigation, kidney function was assessed using a semi-automatic biochemical analyzer, histopathology was examined using Hematoxylin-Eosin staining and immunohistochemistry, and the concentration of inflammatory cytokines was assessed using an enzyme-linked immunosorbent assay (ELISA) test. In addition, using Western blot and q-PCR, the expression of proteins and genes connected to the NF-κB signaling pathway in mice with lipopolysaccharide (LPS)-induced AKI was found. The findings showed that under AKI intervention in LPS group, ACT group and ISO group, the expression of *Rela* (*Rela* gene is responsible for the expression of NFκB p65 protein) and *Tlr4* mRNA was considerably elevated (*P*<0.01), which led to a significant improvement in the expression of MyD88, TLR4, Iκ-Bɑ and NF-κB p65 protein (*P*<0.001). The levels of Alb, Crea and BUN (*P*<0.001) increased along with the release of downstream inflammatory factors such as IL-1β, IL-6, Cys-C, SOD1 and TNF-α (*P*<0.001). More importantly, the study showed that ISO had a more favorable impact on LPS-induced AKI mice than ACT. In conclusion, by inhibiting NF-κB signaling pathway, ACT and ISO could relieve renal failure and inflammation in AKI, offering a fresh possibility for the therapeutic management of the condition.

## Introduction

Acute kidney injury (AKI) is a severe clinical and multifactorial illness characterized by a sudden loss of renal functioning [1]. It is marked by impaired renal tubular reabsorption and decreased glomerular filtration rate [2]. Global statistics show that AKI-induced morbidity and mortality are rather high [3]. According to clinical data, approximately 35% of critical care patients suffer acute kidney failure [4,5]. Intensive care unit (ICU) patients with AKI have a death rate of 60–80% [6,7]. Furthermore, there is evidence that the death rate among individuals with AKI, particularly those requiring dialysis, could reach over 70 percent [8]. AKI patients also encounter a number of complications [9]. AKI is becoming more and more prevalent, and the elevated mortality rate and poor prognosis indicate that it poses a severe health concern [10]. Despite decades of research, the cause of AKI remains unidentified and effective therapies are in short supply. As a result, it is essential to discover novel and potent medications for the treatment of AKI. Animal models that accurately mirror human illnesses are extremely helpful. Gram-negative bacteria infection can now cause renal ischemia, or a deterioration in renal perfusion [11] as well as alterations to renal function [12]. Lipopolysaccharide (LPS) is a biological inducer that has been widely employed for building AKI model [13].

Inflammatory factor storms develop in the early to mid-late stages of AKI. Endotoxin Lipopolysaccharide (LPS) is a traditional ligand for the Toll-like receptor 4 (TLR4). TLR4 has been identified as an LPS receptor. When exposed to LPS, TLR4 triggered the Myeloid differentiation primary response gene 88 (MyD88)-dependent signaling pathway. When LPS activates the TLR4 dimer, it interacts with the downstream adaptor protein MyD88 to cause NF-κB signal transduction [14,15], triggering the NF-κB p65 transcription responses, and inducing secretion of proinflammatory cytokine within dendritic cells, macrophages, and a few epithelial cells [16], for instance, tumor necrosis factor α(TNF-α), interleukin-1β (IL-1β), and interleukin-6 (IL-6), and mediators by activating the nuclear factor-kappa B NF-κB pathway [17]. IL-1β induces both local and systemic inflammation in various autoinflammatory diseases, including allergic asthma, familial mediterranean fever (FMF) and encephalomyelitis (EAE), Tumor necrosis factor-α (TNF-α) is essential for forming a defensive line against pathogenic organisms and has significant effects on the immune system’s normal response by controlling immune cell activation, proliferation, necrosis, and programmed cell death [18,19]. To control the inflammatory pathway and minimize the inflammatory factor storm, administration might be used. Dexamethasone offers therapeutic advantages, but it also has toxic side effects and clinical limitations as demonstrated by studies [20]. Thus, the need for new therapeutic agents is essential.

Acteoside (ACT) and isoacteoside (ISO) are naturally occurring phenylethanoid glycosides that have been isolated from *Cistanche deserticola*, a well-known plant species [21]. Numerous studies have demonstrated pharmacological effects of ACT and ISO, including their antiviral, antioxidant, anti-tumor, anti-inflammatory, hepatoprotective, and immunomodulatory properties [22]. The effects of ACT and ISO on acute kidney injury (AKI) caused by LPS have been reviewed [23,24]. However, the impact and mechanism of ACT and ISO on AKI remain unclear [25]. In this study, we investigated whether ACT and ISO could cure LPS-induced AKI mice, as well as the mechanistic aspects.

## Materials and methods

### 1.1 Animals

A total of 60 specific pathogen Free (SPF) BALB/c mice (Male, 6–8 weeks old,18~22 g) were acquired from Liaoning Changsheng Biotechnology Co. (Production certificate: SCXK(LIAO) 2020–0001). All mice were housed at constant room temperature (25±2°C) and atrelative humidity levels of 60±10%. The circumstance was subjected to 12-hour cycles of darkness and light, and mice were provided unrestricted accessibility to food or water. Before beginning the experiments, the mice were given at least 7 days to get used to these environments.

### 1.2 Drugs and administration

**Fig 1:**
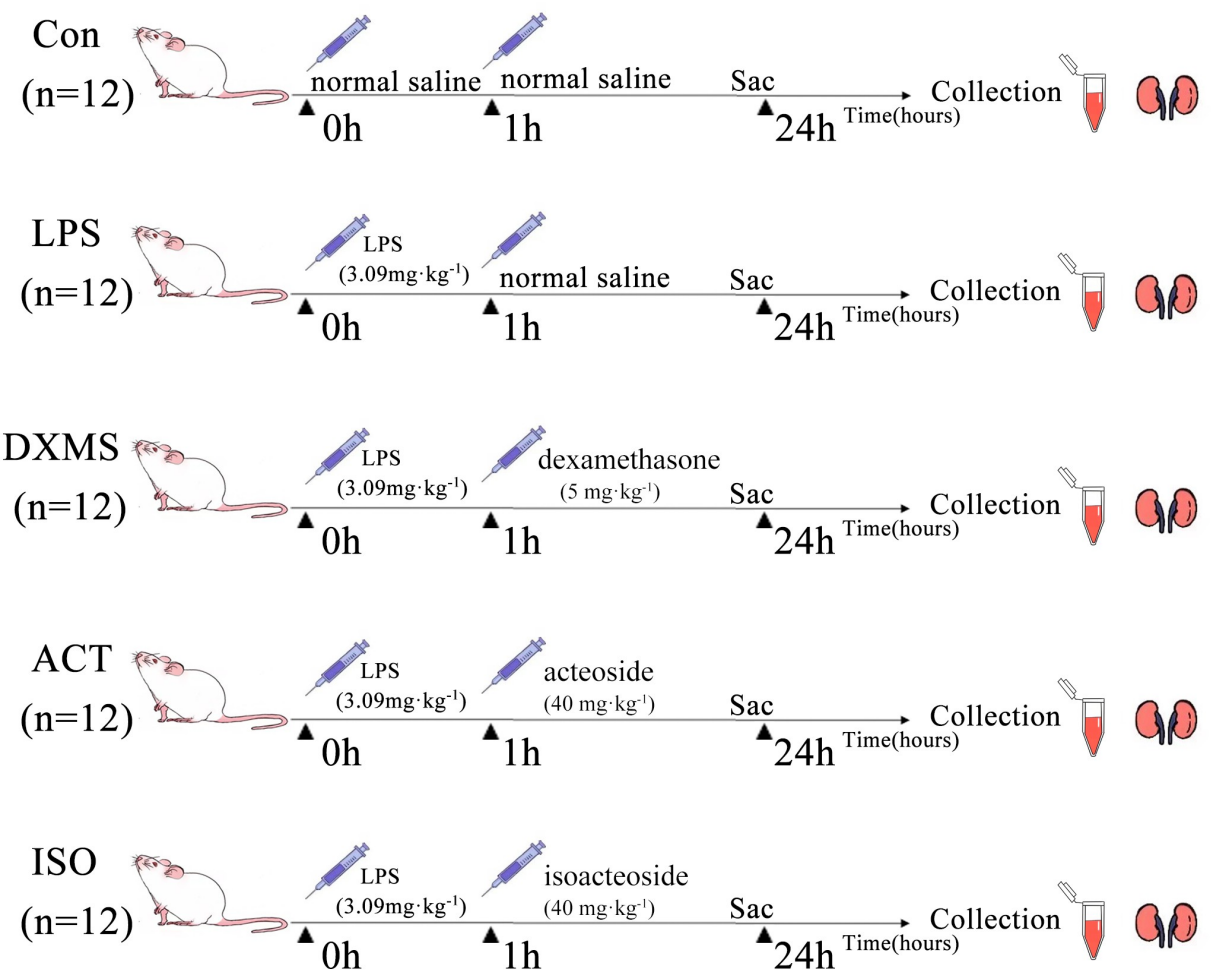
Schematic view of the experimental procedures of the LPS-induced AKI mice model. (Fig 1: Republished from [PLOS ONE] under a CC BY license, with permission from [Jing Lian and Figdraw], original copyright [2024]).

The studyprotocol was reviewed and approved by the ethics regulations of the Animal Care and Use Committee of Liaoning University of TCM IACUC (SYXK [Liao] 2019–0004). Fig 1 depicted the specifics of the animal experiment design. In brief, following a week of acclimatization, the mice were randomly divided into 5 groups of 12 animals each: control group (Con); LPS group (LPS): induction of the AKI model was performed as previously described [26], mice were given a single intraperitoneally injection of Lipopolysaccharides (LPS, 3.09 mg·kg^-1^, L8880, Beijing Solarbio Science & Technology Co. Ltd.) to induce acute kidney injury; positive dexamethasone group (DXMS): Mice were injected intraperitoneally with dexamethasone (5 mg·kg^−1^, DXMS, 20210616, China) 1h after AKI induction; acteoside group (ACT): Mice were injected intraperitoneally with acteoside (Purity≥98%, 40mg·kg^−1^, MUST-20092315, Chengdu Must Bio-Technology (Chengdu, China)) 1h after AKI induction (LPS, 3.09 mg·kg^-1^, injected intraperitoneally); isoacteoside group (ISO): Mice were injected intraperitoneally with isoacteoside (Purity≥98%, 40mg·kg^−1^, MUST-20103104, Chengdu Must Bio-Technology (Chengdu, China)) 1h after AKI induction (LPS, 3.09 mg·kg^-1^, injected intraperitoneally). The selection of dosage is based on the results of pre-experiments and references [23,27].

### 1.3 Sample collection

Twenty-four hours after LPS injection, mice were anesthetized by intraperitoneal injection of 1% pentobarbital sodium and euthanized by cervical dislocation, blood and kidneys were collected for further analysis. The blood was then centrifuged at 3000 g for 20 min to prepare a serum sample. To calculate the kidney index, the kidney was dissected and weighed. And the kidney index of the mice was calculated as:

$$\text{kidney}\;\text{index}\left(\text{\%}\right)=\frac{\text{kidney}\;\text{weight}\left(\text{g}\right)}{\text{Body}\;\text{weight}\left(\text{g}\right)}\times 100$$

Organ tissues were divided into two parts; one part was fixed in 4% formaldehyde for histological analysis, and the other was frozen in liquid N_2_. To determine the biochemical index, all serum and tissue samples were stored at −80°C.

### 1.4 Kidney function test

The serum biochemical index kits for albumin (Alb, 200371), Blood urea nitrogen (BUN, 190451), and creatinine (Crea,21082335N) were bought from Biosino Bio-Technology and Science Inc (Beijing, China). The serum concentrations of Crea, BUN, and Alb were evaluated using a biochemical autoanalyzer (Perlong, Beijing, China).

### 1.5 Hematoxylin-Eosin staining (H&E staining)

Fresh renal tissue was promptly segregated. The fixing of section was done in 4% paraformaldehyde for 24 h before being dehydrated and embedded in paraffin. Xylene was used to dewax the sections, which were later rehydrated in water using a gradient of ethanol. Following dehydration hematoxylin was used to seal the nucleus and cytoplasm for 3 minutes, followed by eosin for 10 seconds before being stained with neutral wax. The histological alterations in the kidneys were graded as elaborated earlier [27]. To avoid repeated scoring of the same H&E staining, we applied the blind method to score the degree of nephropathy injury. The higher the score, the more severe the injury. According to the phenomenon and degree of tubular epithelial cell flattening, loss of brush border, formation of cell membrane bubbles, interstitial edema, cytoplasmic vacuolation, cell necrosis and tubular lumen obstruction, renal tubular epithelial hyperplasia, and loss of brush border, the scoring criteria were divided into five grades: Level 1 is for normal kidney tissue; The area of renal tubular injury with <25% is level 2; The area of renal tubular injury ranging from 25% to 50% is level 3; The area of renal tubular injury ranging from 50% to 75% is level 4; 75%~100% of the renal tubular injury area is level 5.

### 1.6 Enzyme linked immunosorbent assay for evaluation of cytokine levels

The ELISA kits for IL-6 (21071935N), IL-1β (21071933N), Cystatin C (Cys-C) (21071931N), Superoxide Dismutase1(SOD1) (21071936N), and TNF-α (21071930N) were taken from Shanghai Kexing Trading Co. Ltd (Shanghai, China). All operations were carried out according to the manufacturers’ protocols. As we’ve previously described [28]. Briefly, 50 μL standard or sample dilutions were added to a 96-well plate. Then 100 μL of enzyme reagent was added to all the wells. 96-well plate after closing with sealing film was then incubated at 37°C for 1 hour. Each well was aspirated and washed 5 times with 350 μL of 1× Wash Solution. We added 50 μL Chromogen A and Chromogen B to the wells and Colour development at 37°C for 15 minutes away from light. Then, 50 μL Stop Solution was added and the OD was read at 450 nm wavelength of enzymatic microplate reader. The concentration and optical density (OD) of the standard sample were utilized for creating a standard curve, subsequently employed to establish the sample concentration.

### 1.7 Detection of proteins expression related to NF-κB signaling pathway Using Western blot technique

For complete protein extraction, 1 mL protein lysate was added to a 25 mg kidney tissue, which was maintained on ice for 20 min before being subjected to centrifugation at 12 000 rpm in 4°C for half-hour. The supernatant was subsequently gathered and bicinchoninic acid (BCA) Protein assessment Kit (20210428, Beijing Solarbio Science & Technology) was employed to ascertain the protein content. Then, the sample buffer was prepared and sodium dodecyl sulfate-polyacrylamide gel electropheresis (SDS-PAGE) gel electrophoresis was performed. PolyVinylideneFluoride (PVDF) membranes (Millipore, USA) with protein were incubated with different primary antibodies at 4°C overnight. The following primary antibodies were used: rabbit anti-β-actin (AH11286487, Bioss), rabbit anti-TLR4 (BJ12022051, Bioss), rabbit anti-inhibitor of NF-κB alpha (IκB-ɑ) (BAO7267205, Bioss), rabbit anti-NF-κB p65 (BJ07088577, Bioss), rabbit anti-MyD88 (AH10163311, Bioss). The next day, PVDF membranes were incubated with secondary antibody (17A061, Wanlei) at room temperature for 1 h. Following that, the membranes were treated with protein bands and the Enhanced chemiluminescence (ECL) plus western blotting substrate (Thermo Fisher, Waltham, USA) was utilized for visualizing them. Syngene Imaging System (Gene Company, Shenyang, USA) recognized the signal of proteins in membranes. Gray scale analysis with Image J software was used to quantify the results.

### 1.8 Immunohistochemistry staining

Renal tissue was fixed in 4% paraformaldehyde and subsequently sectioned into 10-micrometer thick renal slices after being embedded into paraffin. Paraffin sections were baked at 60°C for 60 min to allow the sections to adhere tightly. Routine dewaxing and rehydration of slices. Washing with phosphate buffered solution (PBS) for two times of 5 min each. Then, a mixture including 1 part of freshly prepared 3% hydrogen peroxide (H_2_O_2_) and 10 parts of distilled water was used to inactivate the endogenous enzyme for 10 min at room temperature. Washing with evaporated water for three times of 2 min each. Overnight incubation of the sections was carried out at 4°C with primary antibody (mouse or rabbit IgG). The objective tissue was covered for 2 h at 37°C in the dark with a secondary antibody (biotinylated goat anti-mouse IgG). Diaminobenzidine (DAB) color development. Staining intensity was observed under a light microscope (Leica Corporation, Germany) and analyzed using ImageJ software. Finally, ImageJ (V1.8.0.112, National Institutes of Health, USA) was used to analyze the positive area in each slide and make statistics.

### 1.9 Polymerase Chain Reaction (PCR)

*Tlr4* and *Rela* (*Rela* gene is responsible for the expression of NFκB p65 protein) expression levels were detected by RT-qPCR. Total RNA was extracted by the Trizol kit (AG RNAex Pro RNA, AG21102, Accurate Biotechnology, Hunan, China) according to the manufacturer’s instructions and the purity of extracted RNA was 1.8–2.2 (OD 260/OD 280). For the removal of genomic DNA:42°C for 2 min then it was kept at 4°C; using reverse transcription kit (Evo M-MLV, AG11728, Accurate Biotechnology, Hunan, China) for the reverse transcription:37°C for 15min, followed by 5 seconds at 85°C, after which it was kept at 4°C, and the RNA was subsequently reverse transcribed into cDNA. The q-PCR reaction system was prepared as follows: 2×SYBR-Green Pro Taq HS Premix (Accurate Biotechnology, Hunan, China) 10 μL, downstream primer (10 μM) 0.4 μL, upstream primer (10μM) 0.4 μL, cDNA template 4μL, Rnase free water 7.2μL. PCR amplification was carried out in an Applied-Biosystems q-PCR machine (Thermo Fisher Scientist, USA). PCR procedure parameters are mentioned below: pre-denaturation at 95°C (30 s), denaturation at 95°C (5 s), and annealing for 30 s at 60°C with 40 cycles in total. The achievements were scrutinized through StepOne Software (version 2.3). *Gapdh*, a housekeeping gene, was employed as the internal reference. Dissolution curve assessment was employed for determining the specificity of PCR amplification, the value of Ct was read, and mRNA target gene relative expression was estimated at the end of the process by the 2^−ΔΔ^Ct technique. Table 1 shows the primer sequences.

**Table 1:** The primers sequences for PCR detection.

Gene	NCBI reference sequence	Sequence	Length (bp)
*Tlr4*	XM_036163964.1	F: 5’-TTCACCTCTGCCTTCACTACA-3’ R:5’-AGACACTACCACAATAACCTTCC-3’	21 23
*Rela* *	NM_001402548.1	F: 5’-CTGGCATCTGTGGACAACTCA-3’ R: 5’-TCACCAGGCGAGTTATAGCTTC-3’	21 22
*Gapdh*	NM_001411843.1	F: 5’-TGTGTCCGTCGTGGATCTGA-3’ R: 5’-TTGCTGTTGAAGTCGCAGGAG-3’	20 21

*Rela gene is responsible for the expression of NFκB p65 protein.

### 1.10 Statistical analysis

The experimental data were analyzed by GraphPad Prism software (version 9.0, San Diego, CA, USA). Each result was presented as the mean ***±*** standard error of the mean (SEM) from at least three independent experiments. Firstly, normality and homogeneity of variance tests were carried out. Then unpaired Student’s t-test were performed for the comparisons between two groups, and one-way analysis of variance (ANOVA) and Tukey’s post hoc test for the comparisons of multiple groups. If the test detection does not conform to the normality and homogeneity of variance, the Dunnett’s T3 test will be used. *P* <0.05 was considered to indicate statistical significance. *P* < 0.01 was considered as significant. *P* < 0.001 were considered as extremely significant.

## Results

### 2.1 ACT and ISO ameliorate renal dysfunction in LPS-induced AKI mice

**Fig 2:**
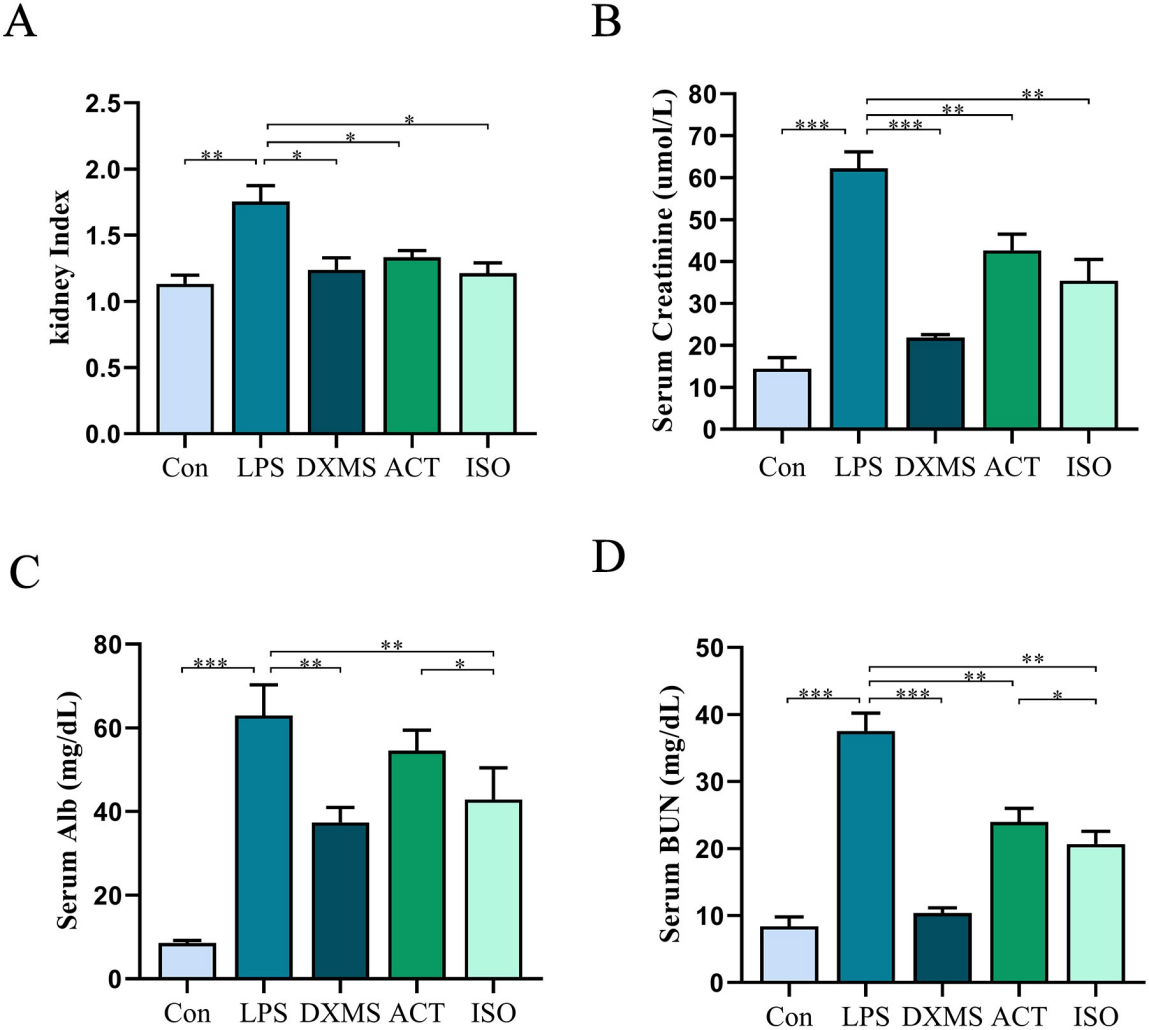
ACT and ISO ameliorate renal dysfunction in LPS-induced AKI mice. (A) Kidney index. (B) Serum creatinine (Crea) concentrations. (C) Serum albumin (Alb) concentration. (D) Blood urea nitrogen (BUN) concentration. Each bar represents the mean ± SEM (n = 6). *p < 0.05 and **p < 0.01, one-way ANOVA with Tukey’s test.

The mice in LPS group showed the symptoms of physical weakness, sluggish responsiveness, lethargy, dull and erect hair, listlessness, reduced urine volume, deepened urine color, no animal suffered hematuria, and several mice developed diarrhea. After receiving lipopolysaccharide, the activity of mice was significantly reduced. However, the DXMS, ACT, and ISO groups of animals had better circumstances as compared to LPS group. The serum concentrations of creatinine, blood urea nitrogen, albumin levels and kidney index were tested to evaluate kidney function [29]. As shown in Fig 2, when comparing the LPS group to the Con group, the kidney index (*P* < 0.01) and the levels of serum for Crea (*P* < 0.001), BUN (*P* < 0.001), and Alb (*P* < 0.001) all showed a substantial rise. While LPS-treated mice that received either ACT or ISO treatment had a distinct reduction in Crea, BUN, and Alb levels as well as kidney index (*P*< 0.05 or *P*< 0.01). Additionally, serum BUN (*P* < 0.05) and Alb (*P* < 0.05) levels were decreased more effectively by ISO than ACT.

### 2.2 ACT and ISO improve pathological changes in LPS-induced AKI mice

**Fig 3:**
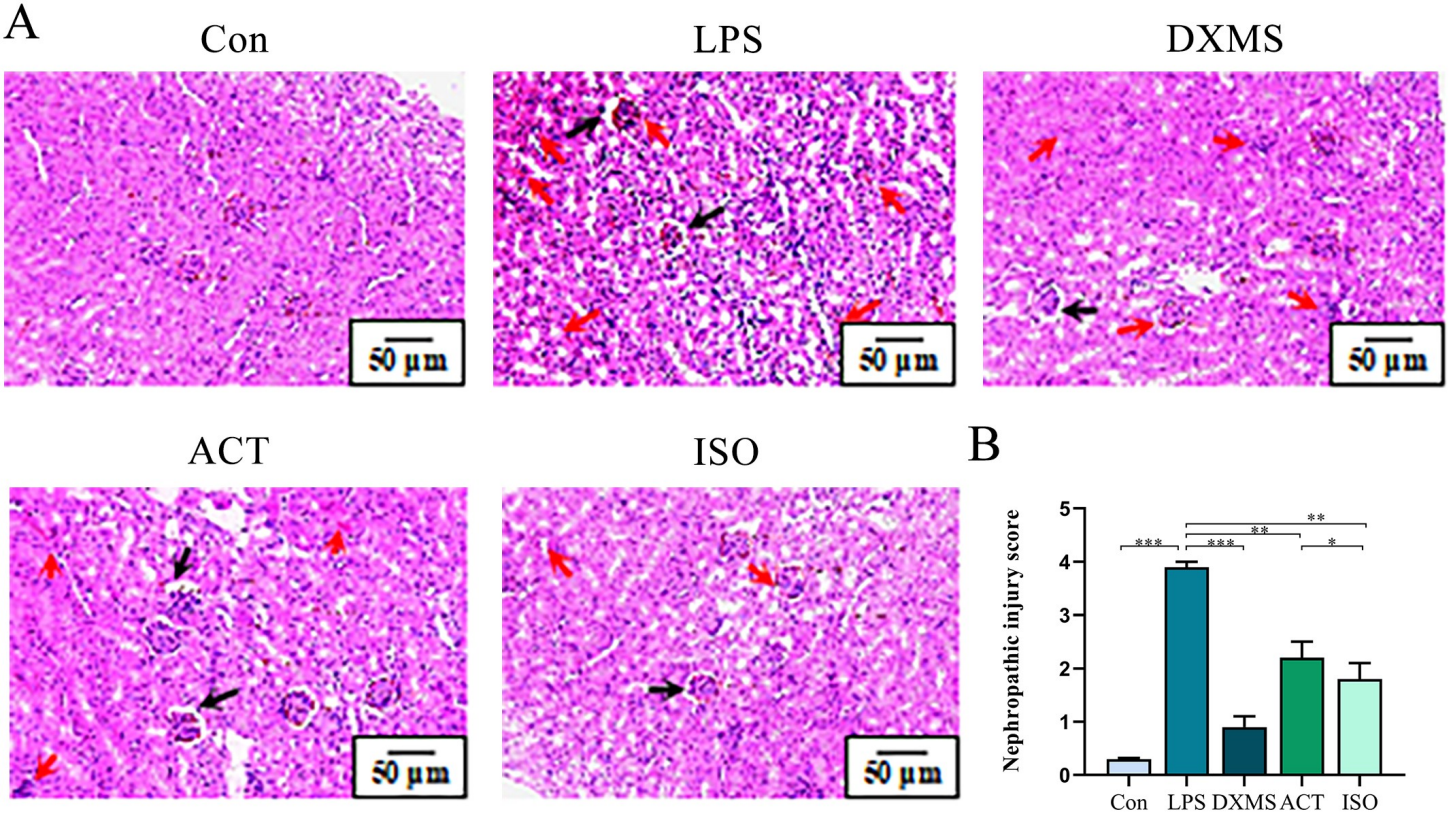
ACT and ISO decrease pathological changes in LPS-induced AKI mice. (A) H&E staining of kidney, red arrows indicate inflammatory cell infiltration and black arrows indicate vacuolar degeneration, scale bar = 50 μm. (B) Nephropathic injury scores. Each bar represents the mean ± SEM (n = 6). *p < 0.05 and **p < 0.01, one-way ANOVA with Tukey’s test.

Results of H&E staining (Fig 3A) showed that mice in the Con group had normal kidney tissue morphology, intact tubular structure, thin and clear walls, with no hemorrhage or inflammatory cell infiltration, as well as uniform glomerular spacing. However, LPS caused tissue damage mainly in the renal cortex and outer medulla. The interstitial cells and stroma showed mild nodular hyperplasia, whereas the tubular epithelium showed massive necrosis, swelling and distortion, vacuolar degeneration, loss of microvilli at the brush border, cell granule degeneration, and nuclear division. As shown in Fig 3B, glomerular cysts and renal cysts were dilated and glomerular hypertrophy and nephropathic injury scores were remarkable increased in the LPS group (*P* < 0.001). ACT (*P* < 0.01) and ISO (*P* < 0.01) groups were less severe than LPS group, and there were significantly different nephropathic injury scores between the ACT and ISO groups (*P* < 0.05), with less inflammatory cell infiltration and erythrocyte exudation, and the improvement of renal pathology in ISO group was better in comparison to that in ACT group.

### 2.3 ACT and ISO improve inflammatory cytokine levels in LPS-induced AKI mice

**Fig 4:**
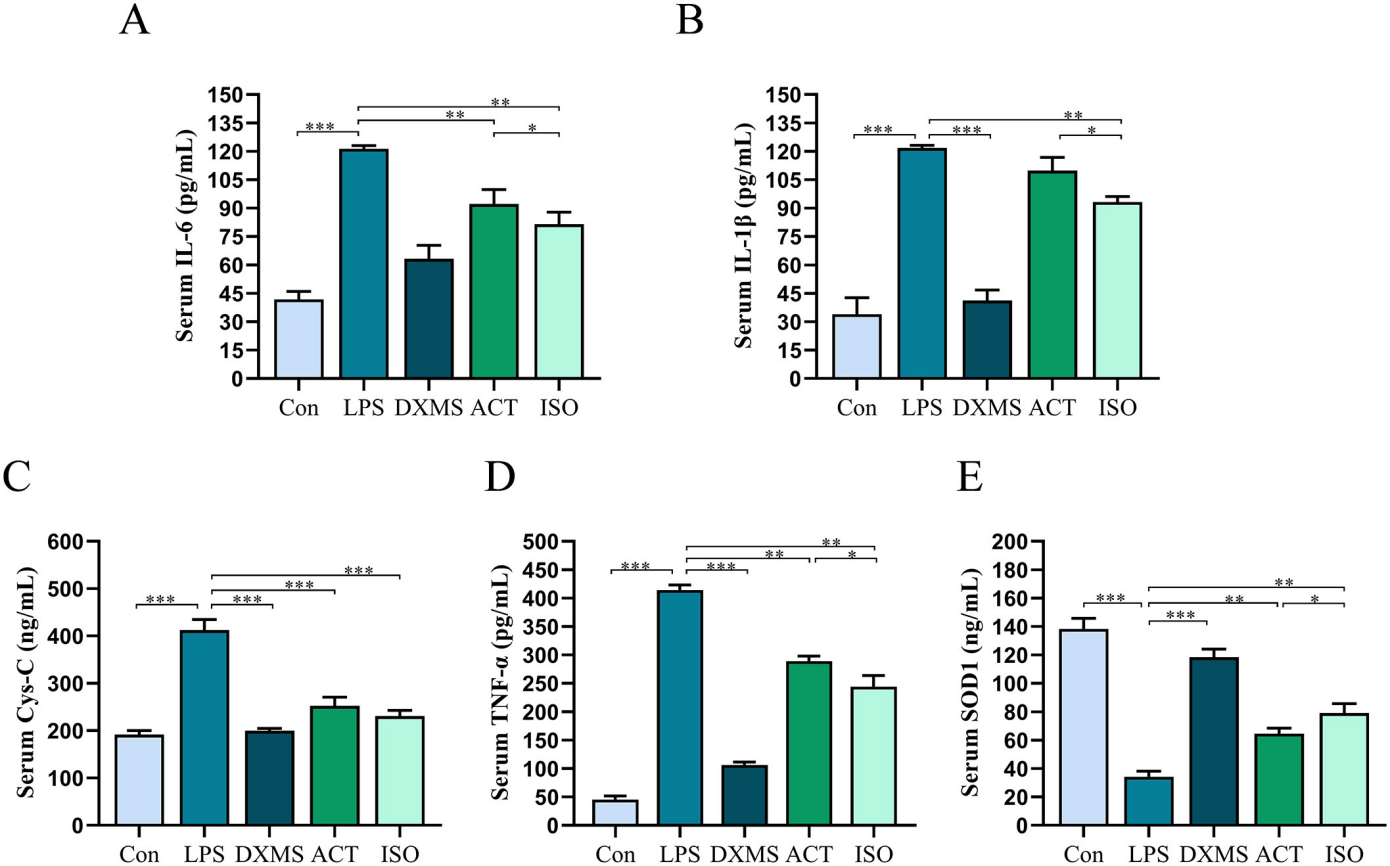
ACT and ISO improve inflammatory cytokine levels in LPS-induced AKI mice. Serum (A)Interleukin-6(IL-6), (B)Interleukin-1β(IL-1β), (C) Cystatin C(Cys-C), (D) Tumor necrosis factor α(TNF-α), (E) Superoxide Dismutase1(SOD1) levels, which reflect renal function, were determined. Each bar represents the mean ± SEM (n = 6). *p < 0.05 and **p < 0.01, one-way ANOVA with Tukey’s test.

Elevated concentration of pro-inflammatory factors is a biological and clinical indicator characterizing acute kidney injury and has an indispensable part in the exacerbation of acute kidney injury [30]. As shown in Fig 4, Serum TNF-α, IL-6, IL-1β, Cys-C, SOD1 had a comparatively higher level in LPS group than Con group (*P*<0.001), suggesting that the AKI model had been successfully established. Fortunately, both ACT and ISO, to varied degrees, reversed the aforementioned alterations in inflammatory factor levels. In addition, we observed that the favorable impact of ISO was more substantial than that of ACT, particularly in the IL-6 (*P*<0.05), IL-1β(*P*<0.05), TNF-α(*P*<0.05) and SOD1 (*P*<0.05) indicators.

### 2.4 ACT and ISO suppress protein and gene expression of TLR4 and NF-κB in LPS-induced AKI mice

**Fig 5:**
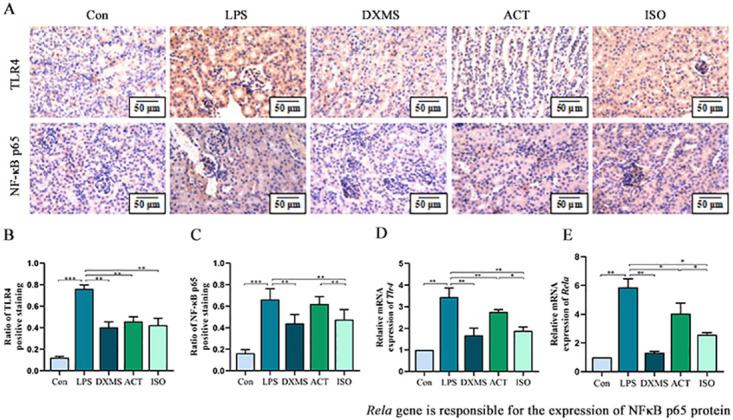
ACT and ISO suppress protein and gene expression of TLR4 and NF-κB in LPS-induced AKI mice. (A) The protein expression of TLR4 and NF-κB p65 tested using immunostaining. Quantitative data of TLR4-positive (B) and NF-κB p65-positive (C) areas. The gene expression of *Tlr4* (D) and *Rela* (*Rela* gene is responsible for the expression of NFκB p65 protein) (E) assessed by q-PCR and the data show quantitative results normalized to GAPDH. Each bar represents the mean ± SEM (n = 6). *p < 0.05 and **p < 0.01, one-way ANOVA with Tukey’s test.

Immunohistochemistry and q-PCR were used to examine the expression of inflammation-related proteins NF-κB p65 and TLR4, and their respective genes, *Rela* and *Tlr4*. As shown in Fig 5A, under the microscope, the positive expression sites of two proteins could be observed, with only a small number of brownish-yellow granules expressed in Con group, thereby implying that normal renal tubular epithelial cells could express a little quantity of NF-κB p65 and TLR4. In the LPS group, the expression of brownish-yellow granules protruding from the background was substantially elevated, as was the mean optical density value, indicating that TLR4 and NF-κB p65 were over-expressed. But ACT, ISO, and DXMS all have the potential to significantly lower the positive expression of these two proteins. Moreover, as compared to the ACT group, the ISO group’s expression of NF-κB p65 was considerably diminished (*P*<0.01), although TLR4 protein expression was not unaffected. Similar to this, q-PCR results revealed that compared with the LPS group, ACT(*P*<0.01) and ISO(*P*<0.01) decreased the elevation of *Tlr4* mRNA expression caused by LPS. Moreover, ISO is more effective than ACT(*P*<0.05). In the case of *Rela* (*Rela* gene is responsible for the expression of NFκB p65 protein), ACT (*P*<0.05) and ISO (*P*<0.05) differentially reduced the mRNA expression compared with the LPS group. In a similar vein, ISO was more effective than ACT (*P*<0.05).

### 2.5 ACT and ISO alleviate renal dysfunction and inflammation via inhibiting NF-κB signaling pathway

**Fig 6:**
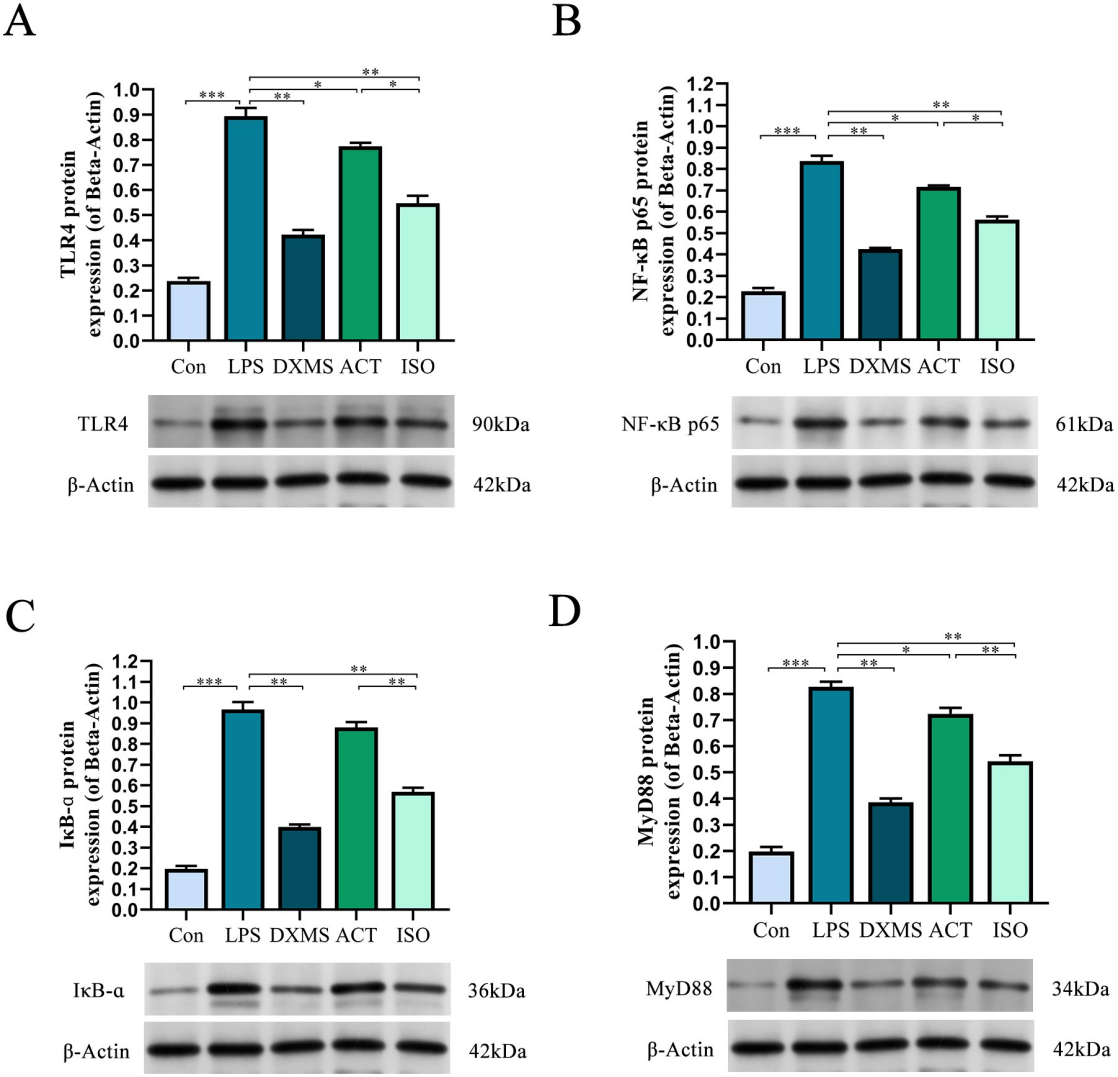
ACT and ISO alleviate renal dysfunction and inflammation via inhibiting NF-κB signaling pathway. The protein expression of TLR4 (A), NF-κB p65 (B), MyD88 (C) and IκB-ɑ (D) detected by Western blot. β-Actin was also analyzed as a loading control. Each bar represents the mean ± SEM (n = 6). *p < 0.05 and **p < 0.01, one-way ANOVA with Tukey’s test.

NF-κB signaling pathway is the classical pathway to study inflammation, and results of immunohistochemistry and q-PCR suggest that ACT and ISO could influence TLR4 and NF-κB p65 expression. On this basis, we intended to use western blot to identify changes in the expression of proteins relevant to the pathway. As shown in Fig 6, our results indicated that, in comparison to the Con group, the expression of TLR4(*P*<0.001), MyD88(*P*<0.001), NF-κB p65(*P*<0.001), and IκB-ɑ(*P*<0.001) proteins was significantly elevated following LPS administration. When compared to the ACT group, ISO effectively down-regulated the levels of IκB-ɑ and MyD88 proteins (*P*<0.01). In addition, ISO was more effective than ACT (*P*<0.05) in regulating the TLR4 and NF-κB p65 proteins, indicating that both ACT and ISO could effectively attenuate the activation of TLR4 / NF-κB pathway.

## Discussion

Acute kidney injury (AKI), also recognized as acute renal failure (ARF), is the abrupt collapse of renal function. AKI, as a systemic illness, is a cluster of syndromes that include urinary tract obstruction [31], sepsis [32], heart failure, cardiorenal syndrome [33], and hepatorenal syndrome, all of which have been shown to be associated with significant morbidity and mortality [34]. AKI has been reported in more than 50% of patients in intensive care and affects around 10–15% of hospitalized patients, and AKI rates in specialist hospital departments such as transplant centers, intensive care units, oncology, and cardiac surgery can reach 50% or higher [35]. AKI pathophysiology can range from a reduction in glomerular filtration rates (GFR) caused exclusively by local or systemic hemodynamic alterations caused by reversible tubular stress or injury to frank tubular necrosis, which results in an increase in serum Crea and BUN levels as well as proteinuria [36]. There is no pharmaceutical treatment for reversing renal damage that has been tried in clinical trials to yet. AKI cannot be avoided or treated with particular medications or therapies. Natural products have been demonstrated in several studies to be safe and effective, which can shorten the time and expense involved in developing new drugs. Furthermore, there is growing scientific interest in the research on natural products for the prevention and treatment of a variety of ailments [37]. Thus, with this medical circumstance. Natural products prevention is always needed. Some functional dietary components have been indicated to sustain renal function, implying that long-term administration of these components might be an effective method to avoid AKI.

Cistanches Herba (*Rou cong rong* in Chinese), is a very precious, tonic traditional Chinese medicine. *Chinese Pharmacopoeia* (2020 edition) records its functions as tonifying kidney yang, benefiting essence and blood, moistening the intestines and relieving constipation. It mainly cures deficiency of kidney yang, absence of essence and blood, impotence and infertility, lumbar and knee soreness, muscle and bone weakness, and intestinal dryness and constipation [38]. The ACT and ISO are phenylethanol glycoside compounds extracted from *Cistanche deserticola* (CD). CD that has been steamed in rice-wine for 16 hours is referred to rice-wine processed CD. In accordance with our earlier investigations, rice-wine processed CD enhances and recovers the function of the hypothalamus’ nerve cells while also promoting immunological function [28]. We found the content of ACT decreased in rice-wine processed CD, while the content of ISO increased detected by HPLC (High Performance Liquid Chromatography) [39]. It has been reported that ACT has certain pharmacological potency against alcohol-induced chronic liver injury and has strong hepatoprotective activity [40], which can prevent liver fibrosis [41]. It can also inhibit the formation of superoxide free radicals and enhance their antioxidant effects [42]. ISO has hepatoprotective effect [43] and protective effect on UV-induced skin photodamage [44]. However, there is limited research on the application of ACT and ISO in the treatment of acute kidney injury.

The TLR4/NF-κB signaling pathway is regarded as a typical pro-inflammatory signaling pathway, known as the switch to initiate and stop inflammation [45]. The bacterial endotoxin LPS functions as a TLR4 ligand, activating the TLR4 cell signaling pathway, which leads to a downstream signal via a MyD88-dependent signaling cascade following identification of the stimulus by TLR4. When MyD88 is actived, it can phosphorylate the specific location of IKKβ and IKKα, leading to IκB phosphorylation, followed by ubiquitination and protease degradation. Once the structure of inhibitory protein changes, it will be separated from NF-κB, resulting in NF-κB inhibition being relieved, while the inhibitory protein is activated to translocate into the nucleus causing excessive expression of inflammatory factors, such as cytokines TNF-α, IL-1β, and IL-6, to generate an inflammatory response [46]. These inflammatory cytokines may activate NF-κB, amplify the initial inflammatory signal, and trigger a vicious cycle that exacerbating the inflammatory response and further damaging the microcirculation of the body [47,48].

**Fig 7:**
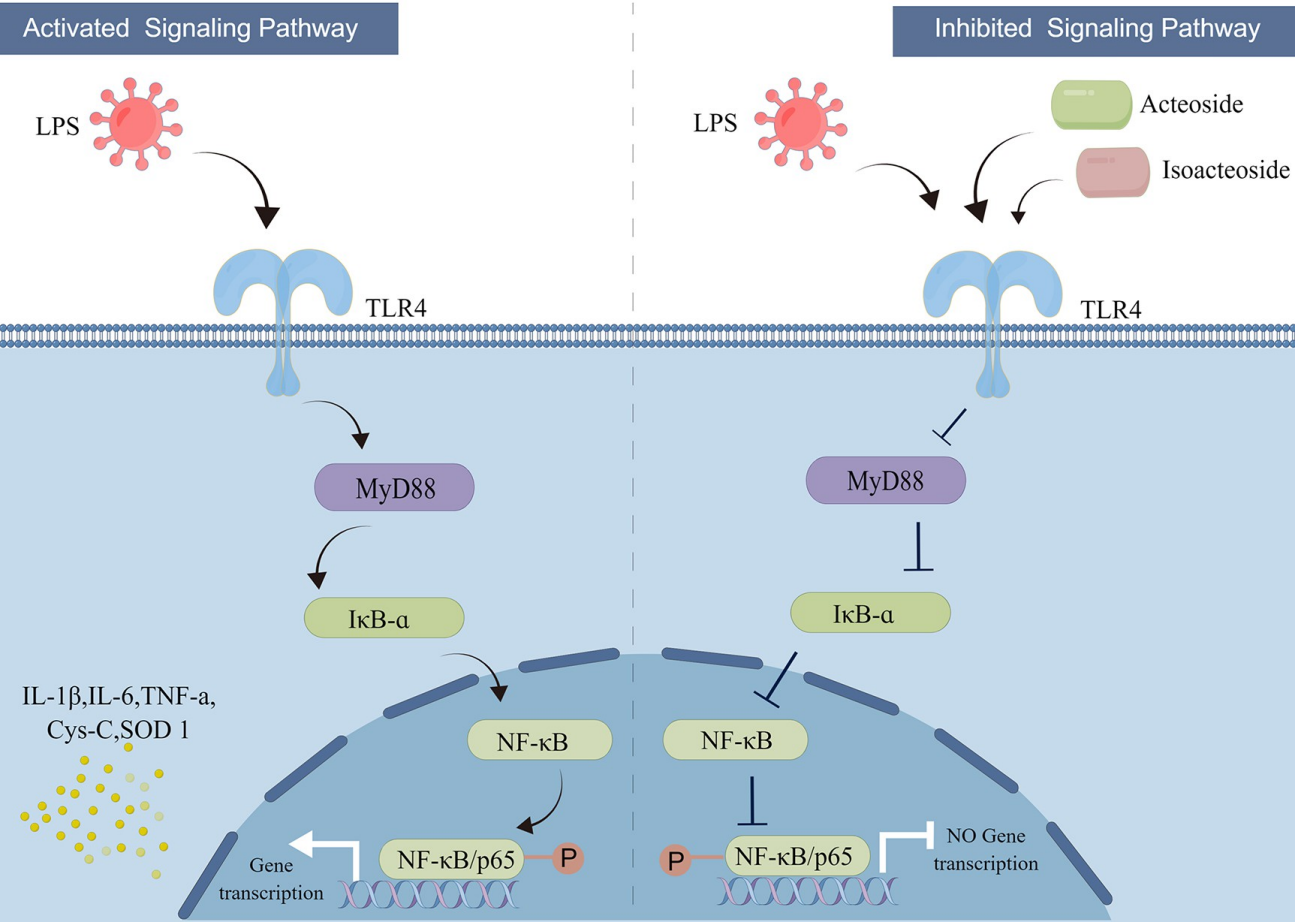
Schematic diagram of the underlying mechanism of acute kidney injury caused by LPS. Acteoside and isoacteoside block the activation of the TLR4/NF-κB signal pathway, thereby impairing the release of pro-inflammatory cytokines. (Fig 7: Republished from [PLOS ONE] under a CC BY license, with permission from [Jing Lian and Figdraw], original copyright [2024]).

LPS can cause acute kidney injury in mice, as demonstrated by an abrupt rise in serum levels and characteristic histological alterations [49]. In the present study, we discovered that the expressions of downstream inflammatory factors of the TLR4/MyD88/NF-κB signal axis, such like IL-1β, IL-6, Cys-C, SOD1 and TNF-α, as well as Alb, Crea, and BUN levels increased appreciably after the AKI processing in the LPS group. Moreover, *Rela* (*Rela* gene is responsible for the expression of NFκB p65 protein) and *Tlr4* mRNA expression was enormously elevated, thereby causing MyD88, TLR4, Iκ-Bɑ, and NF-κB p65 proteins expression increased significantly. The ACT and ISO greatly lowered the serum levels of Crea, BUN and Alb, which are inflammatory mediators produced by LPS. ACT and ISO also reduced the release of inflammatory factors (IL-1β, IL-6, Cys-C, SOD1, TNF-α). Histopathological examination demonstrated that the therapeutic effect of ISO was superior to that of ACT, providing sufficient proof that the agent is capable of alleviating AKI in mice. By targeting on the TLR4 / NF-κB axis to avert LPS-induced AKI response, ACT and ISO inhibited the transcription and translation of inflammatory genes (*Rela* and *Tlr4*) and proteins (TLR4, NF-κB, MyD88, IκB-ɑ) expression. Taken together, our findings suggest that the phenylethanol glycosides AKI and ISO isolated from CD have superior efficacy in the TLR4/MyD88/NF-B signaling pathway for the treatment of AKI (Fig 7). Additionally, the effect of ISO is stronger to that of ACT, indicating that rice-wine processed CD is more effective than unprocessed CD in tonifying kidney yang. Our future experiments will explore other possible pathways and related factors on the pathways to analyze the mechanism of anti-acute kidney injury of ACT and ISO, such as *Slc5a2*, *Lcn2* and *Havcr1*, meanwhile, in the next study, we will also compare the ISO group with the ISO+ACT synergistic group, to further prove that the ISO has a better pharmacological effect in the treatment of acute kidney injury.

## Supporting information

S1 Fig Original uncropped images for Western blot. (TIF)

S2 Fig The result of Western blot repeat experiments. The protein expression of TLR4, NF-κB p65, MyD88 and IκB-ɑ detected by Western blot. β-Actin was also analyzed as a loading control.
(TIF)

S3 Fig The result of Western blot repeat experiments. The protein expression of TLR4, NF-κB p65, MyD88 and IκB-ɑ detected by Western blot. β-Actin was also analyzed as a loading control.
(TIF)

## Abbreviations

ACT: Acteoside
AKI: Acute kidney injury
Alb: Albumin
BCA: Bicinchoninic acid
BUN: Blood urea nitrogen
Crea: Creatinine
Cys-C: Cystatin C
DXMS: Dexamethasone
EAE: Encephalomyelitis
ELISA: Enzyme-linked immunosorbent assay
FMF: Familial mediterranean fever
GFR: Glomerular filtration rates
IL-1β: Interleukin-1β
IL-6: Interleukin-6
ISO: Isoacteoside
LPS: Lipopolysaccharide
MyD88: Myeloid differentiation primary response gene 88
PBS: Phosphate buffered solution
PCR: Polymerase Chain Reaction
PVDF: PolyVinylideneFluoride
SOD1: Superoxide Dismutase1
TLR4: Toll-like receptor 4
TNF-α: Tumor necrosis factor α
