# Supplementary figures and images for: Acteoside and isoacteoside alleviate renal dysfunction and inflammation in lipopolysaccharide-induced acute kidney injuries through inhibition of NF-κB signaling pathway
Source: PLoS One. 2024 May 15;19(5):e0303740. doi: 10.1371/journal.pone.0303740 (PMC11095724; doi:10.1371/journal.pone.0303740)

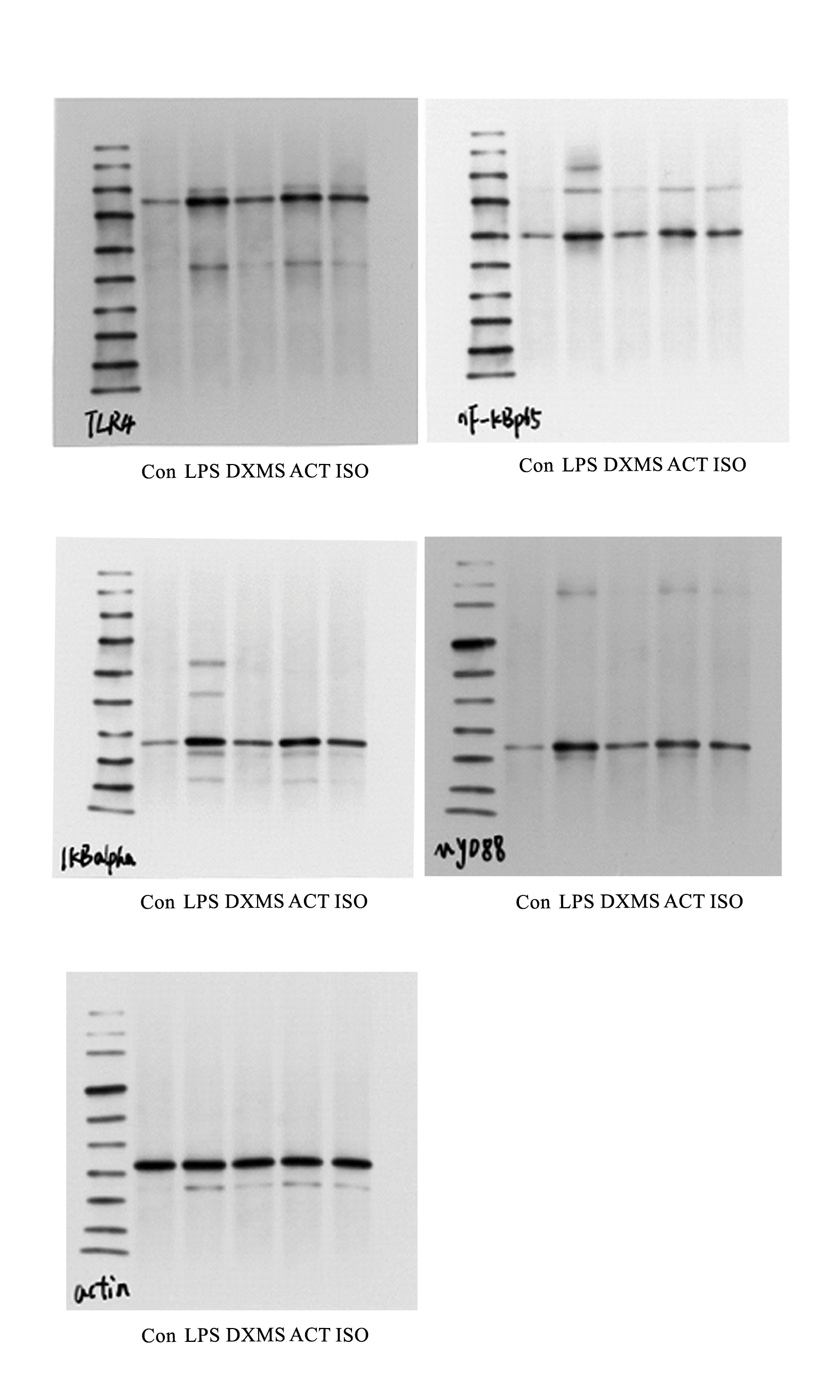

Supplement: S1 Fig — (TIF) [file pone.0303740.s001.tif]

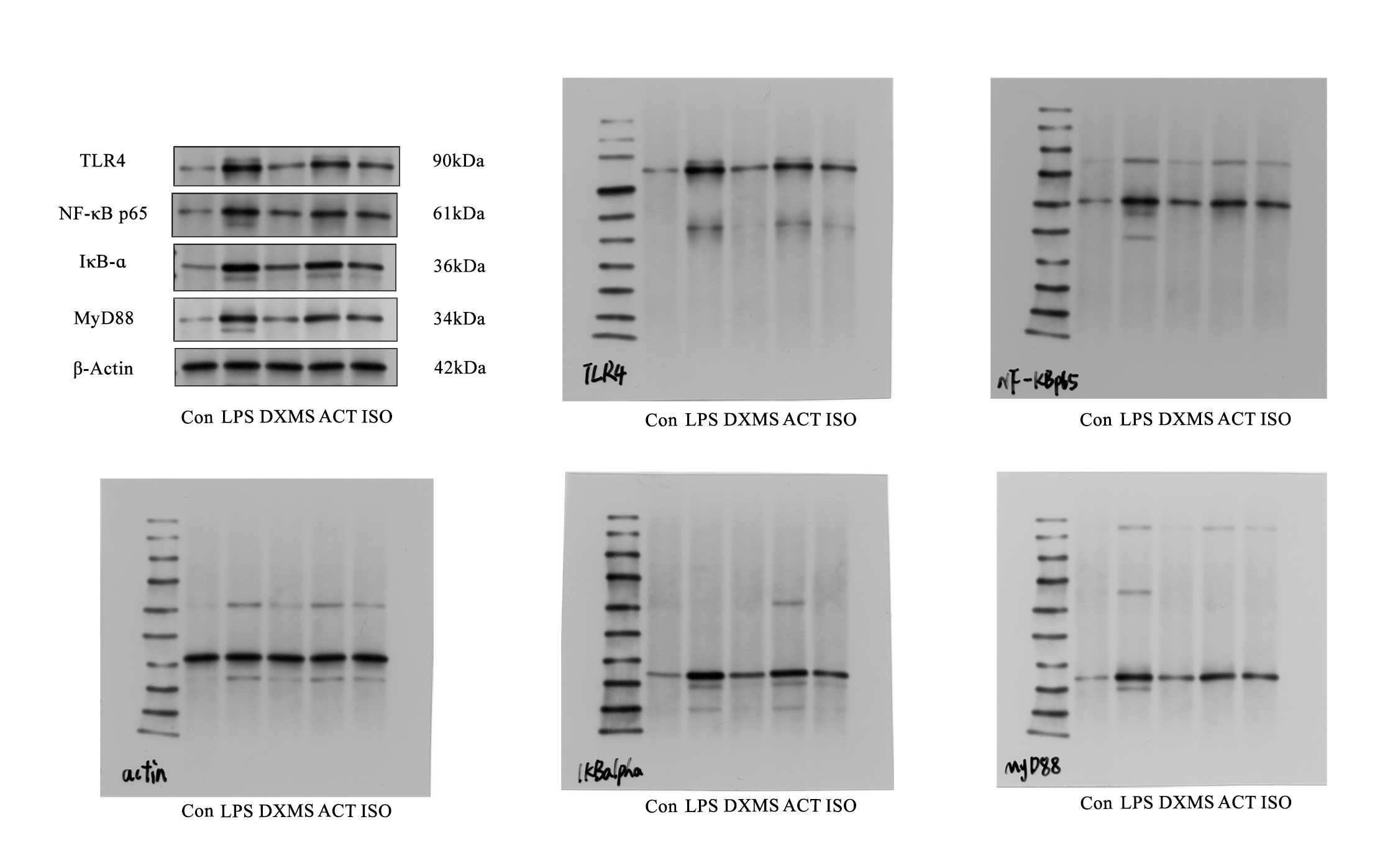

Supplement: S2 Fig — The protein expression of TLR4, NF-κB p65, MyD88 and IκB-ɑ detected by Western blot. β-Actin was also analyzed as a loading control. (TIF) [file pone.0303740.s002.tif]

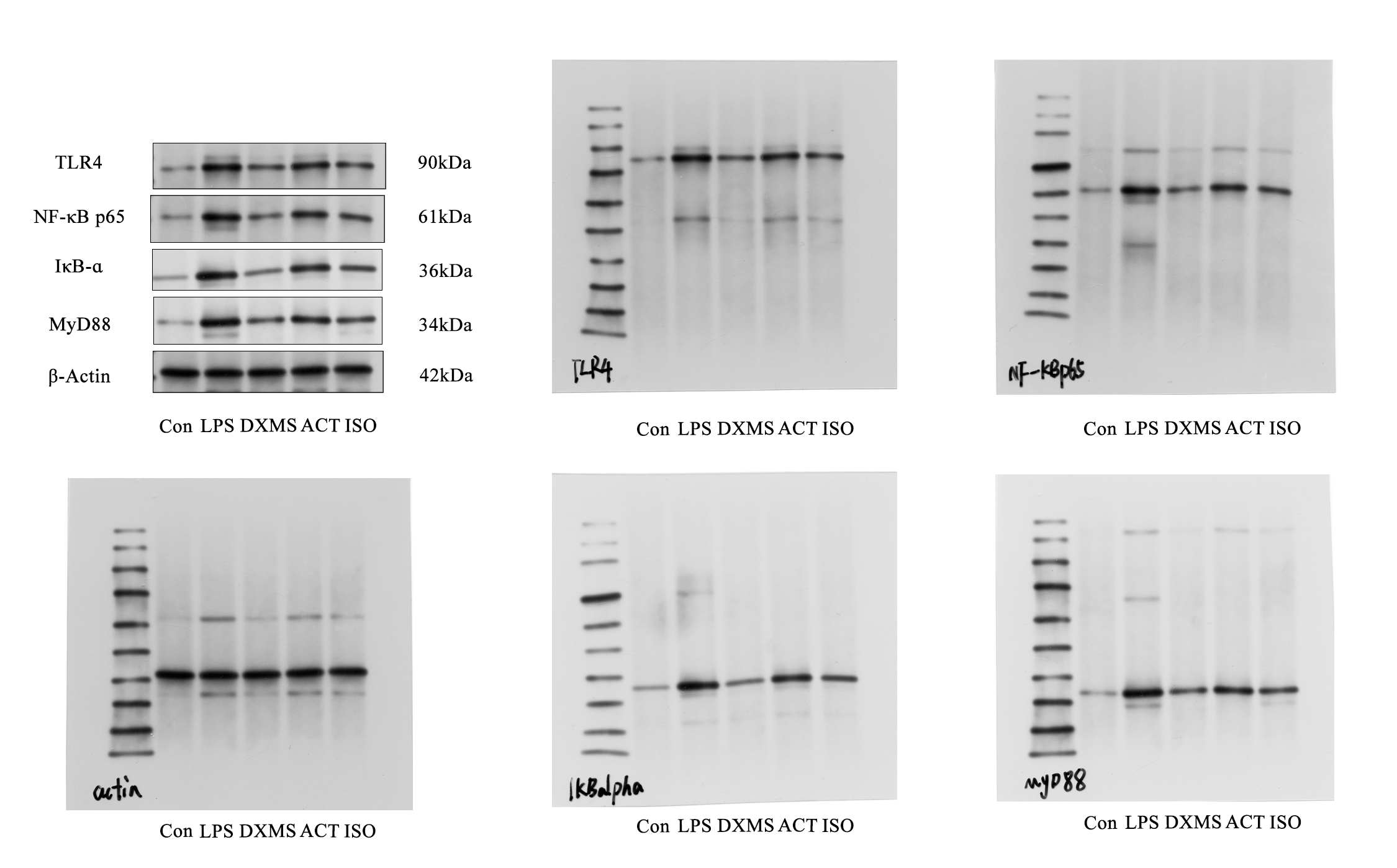

Supplement: S3 Fig — The protein expression of TLR4, NF-κB p65, MyD88 and IκB-ɑ detected by Western blot. β-Actin was also analyzed as a loading control. (TIF) [file pone.0303740.s003.tif]
